# Molecular and Cytogenetic Analysis of rDNA Evolution in Crepis Sensu Lato

**DOI:** 10.3390/ijms23073643

**Published:** 2022-03-26

**Authors:** Magdalena Senderowicz, Teresa Nowak, Hanna Weiss-Schneeweiss, Laszlo Papp, Bozena Kolano

**Affiliations:** 1 Institute of Biology, Biotechnology and Environmental Protection, Faculty of Natural Sciences, University of Silesia in Katowice, 40-007 Katowice, Poland; senderowicz.magdalena@gmail.com (M.S.); teresa.nowak@us.edu.pl (T.N.); 2 Department of Botany and Biodiversity Research, University of Vienna, Rennweg 14, A-1030 Vienna, Austria; hanna.schneeweiss@univie.ac.at; 3 Eötvös Loránd University Botanical Garden, Illés u. 25, 1083 Budapest, Hungary; papplaca@gmail.com

**Keywords:** rDNA loci, Crepis, 5S rDNA NTS, nrITS, chromosomes, FISH, phylogeny

## Abstract

Although Crepis was the first model plant group in which chromosomal changes were considered to play an important role in speciation, their chromosome structure and evolution have been barely investigated using molecular cytogenetic methods. The aim of the study was to provide a better understanding of the patterns and directions of Crepis chromosome evolution, using comparative analyses of rDNA loci number and localisation. The chromosome base number and chromosomal organisation of 5S and 35S rDNA loci were analysed in the phylogenetic background for 39 species of Crepis, which represent the evolutionary lineages of Crepis sensu stricto and Lagoseris, including *Lapsana communis*. The phylogenetic relationships among all the species were inferred from nrITS and newly obtained 5S rDNA NTS sequences. Despite high variations in rDNA loci chromosomal organisation, most species had a chromosome with both rDNA loci within the same (usually short) chromosomal arm. The comparative analyses revealed several independent rDNA loci number gains and loci repositioning that accompanied diversification and speciation in Crepis. Some of the changes in rDNA loci patterns were reconstructed for the same evolutionary lineages as descending dysploidy.

## 1. Introduction

Changes in the karyotype structure, which often accompany diversification and speciation events, are a focal point of plant evolutionary studies [1,2,3]. Chromosomal rearrangements are not only associated with changes in the size and structure of chromosomes but also with changes in the chromosome base numbers via ascending or descending dysploidy [1,2,4]. The dysploid chromosomal changes have been inferred for many species of Asteraceae [5] and other plant families (e.g., Fabaceae [6]). The patterns and mechanisms of karyotype evolution have been most extensively studied in two plant families that include model species, Poaceae (e.g., *Brachypodium dystachion* and *Oryza sativa*) [3,7,8,9] and Brassicaceae (*Arabidopsis thaliana* [10,11,12]). Analyses of the localisation of the BACs (bacterial artificial chromosomes) using FISH (fluorescent in situ hybridisation) has demonstrated that different types of chromosomal rearrangements, which are often group-specific, accompanied the evolution of their karyotypes. Inversions and translocations were detected as the most frequent types of chromosomal rearrangements in the Brassicaceae family [11], whereas, in Brachypodium (Poaceae), descending dysploidy has been inferred to have occurred primarily via nested chromosomal fusions [7,8]. The use of FISH with oligo probes that are specific for selected regions of individual chromosomes enabled the occurrence of the reciprocal chromosomal translocations that accompanied the evolution and speciation of two Solanum species to be demonstrated [13]. These approaches, however, can only be used in taxa, for which whole-genome assemblies for at least one member of the analysed group is available [7,14]. In non-model species, comparative analyses of chromosome structure and karyotype evolution usually rely on FISH with various repetitive sequences as the probes [15,16]. Comparative mapping of chromosomal markers might enable at least some chromosomal rearrangements to be identified and thus enable a better understanding of the patterns of their chromosomal evolution [17,18]. 5S and 35S rDNAs are most often used as chromosomal markers, as they are highly repetitive, arranged in tandem arrays and highly conserved in the DNA sequence of the coding regions [19,20,21,22,23]. The non-transcribed spacer (NTS) of 5S rDNA and the internal transcribed spacer of 35SrDNA (nrITS 1 and 2) evolve much faster than their coding regions and are thus often used as molecular markers in phylogenetic analyses [24]. In most higher plants, the number and localisation of the 35S and 5S rDNA loci are unlinked [25].

Crepis species are mostly diploids with chromosome base numbers of x = 3, 4, 5, 6 and 11. Three evolutionary lineages were identified in Crepis sensu lato (s.l): (i) Crepis species with a chromosome base number x = 7, now the genus Askellia; (ii) Lagoseris, which encompasses several Crepis species (e.g., *C. palaestina* and *C. praemorsa*) and two other lineages, which are now classified as the genera Lapsana and Rhagadiolus, and (iii) Crepis sensu stricto (Crepis s.s. [26,27]). The chromosomes of Crepis species are relatively large and well-differentiated within the karyotype [27,28]. Analyses of the chromosome numbers and karyotype structure in phylogenetic framework inferred x = 6 as an ancestral state for Crepis s.l. Several subsequent and independent descending dysploidy events led to the evolution of derived chromosome base numbers (x = 5, 4, 3) during the diversification of the genus [27]. Most of the currently accepted clades of closely related Crepis species include taxa that differ in chromosome numbers and karyotype morphology [26,27].

Molecular cytogenetic analyses of Crepis karyotypes are scarce and usually encompass fluorochrome banding and the localisation of a few repetitive DNA sequences using FISH. The chromosomal organisation of two satellite DNA sequences (pCcD32 and pCcE9) has only been analysed in the *C. capillaris* genome (2n = 6) and revealed chromosome-specific hybridisation patterns of these two repeats [29]. The chromosomal organisation of the rRNA gene loci was studied in *C. capillaris* (2n = 6) and three closely related species from the section Neglectoides: *C. neglecta* (2n = 8), *C. cretica* (2n = 8) and *C. hellenica* (2n = 6 [30]). In *C. capillaris*, one locus of each of the 35S and 5S rDNAs were observed [31,32]. Both rDNA loci were located in the short arm of the subtelocentric chromosome, with the 35S rDNA locus in a more distal position. In the other three species, to the subtelocentric chromosome that carried both the 35S and 5S rDNA loci and an additional second locus of 35S rDNA in the pericentromeric region of another chromosome were observed [30].

The aims of this study were to analyse the chromosomal organisation and evolution of the rDNA loci in 39 Crepis species belonging to the Crepis s.s. and Lagoseris evolutionary lineages; the latter also includes *Lapsana communis*. The specific objectives were to (1) establish the number and localisation of the 5S and 35S rDNA loci in all of the analysed species, most for the first time, (2) to reconstruct the ancestral states of the 5S and 35S rDNA loci numbers for the genus and (3) to infer the patterns and directionality of the rDNA loci evolution within the phylogenetic framework that is inferred from the analyses of the nrITS [27]. Double fluorochrome banding with CMA_3_ and DAPI was used to identify the relationships between the localisation of rDNA loci and the positive CMA_3_ bands. The newly generated 5S rDNA sequence data gave insight into the intra- and interspecific polymorphism of the 5S rDNA sequences.

## 2. Results

### 2.1. Phylogenetic Analyses of the 5S rDNA NTS and nrITS

**Figure 1:**
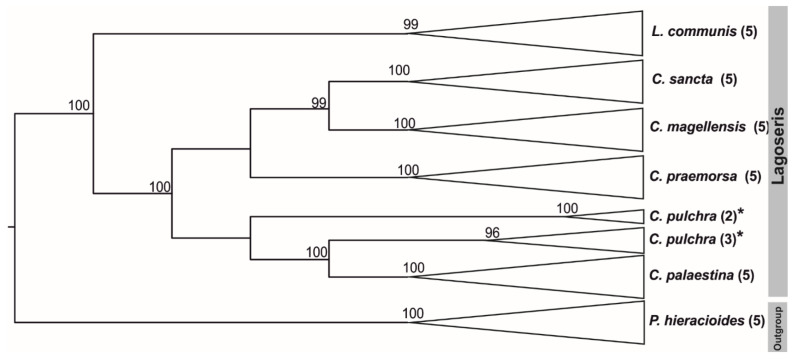
Phylogenetic analysis of cloned sequences of the 5S rDNA non-transcribed spacer representing species of Lagoseris lineage. Numbers in parentheses indicate the number of analysed clones. The tree was rooted with *Picris hieracioides*. Bootstrap scores are shown above branches. The stars indicate species with two variants of 5S rDNA NTS.

Phylogenetic analyses of the 5S rDNA NTS were conducted separately for the Lagoseris and Crepis s.s. lineages due to the high levels of sequence divergence. The length of the analysed region ranged from 252 to 353 bp among all of the analysed Crepis species and from 673 to 677 bp in *L. communis*. The alignment of the Lagoseris sequences was 738 bp long with 192 parsimony informative sites, whereas the alignment of the Crepis s.s. sequences was 446 bp long with 364 parsimony informative sites. The inferred phylogenetic relationships revealed two main groups in Lagoseris (Figure 1 and Figure S1). The first group consisted solely of *L. communis* (BS99), whereas the second comprised the remaining five species of this lineage. These species formed three groups that were identified as: (i) a clade consisting of *C. sancta* and *C. magellensis* (BS99), (ii) a clade consisting of *C. praemorsa* (BS100) and (iii) a clade consisting of *C. palaestina* and *C. pulchra* (Figure 1).

**Figure 2:**
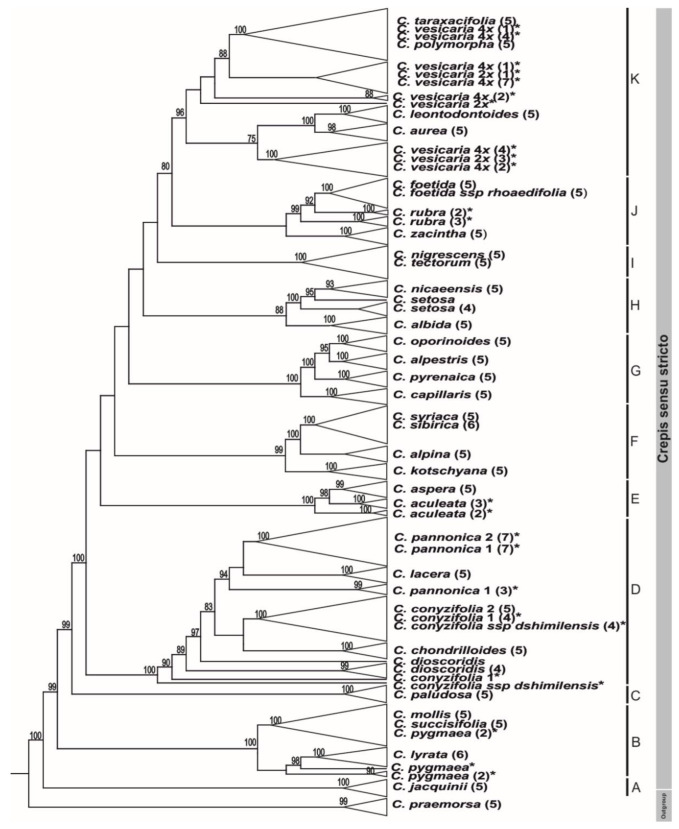
Phylogenetic analysis of the cloned sequences of 5S rDNA non-transcribed spacer representing species of Crepis s.s. lineage. Numbers in parentheses indicate the number of clones analysed. The tree was rooted with *C. praemorsa*. Bootstrap scores are shown above branches. Stars indicate species with two variants of 5S rDNA NTS. A–K letters indicates clades. In the ML analysis of the nrITS region, two main well-supported evolutionary lineages were recovered: Lagoseris (BS94) and Crepis s.s. (BS85; Figure 3). In Crepis s.s., four main clades (1–4) that had high bootstrap support (BS72-BS100) were found. The newly sequenced *C. chondrilloides* was recovered in clade 3 (Figure S3).

All of the analysed species of the Crepis s.s. lineage were assigned to 12 clades (Figure 2 and Figure S2). Most of the clades were well-supported, whereas most of the nodes in the backbone of the tree were poorly supported (Figure 2). The clades that were recovered from the 5S rDNA NTS analyses are labelled with capital letters (clades A–K). The first three well-supported clades (clades A–C, all BS100) comprised species with x = 6. Clade D (BS100) primarily consisted of perennial species with x = 4 (Figure 2 and Figure S2). The remaining clades (E–K) consisted of species with chromosome base numbers of x = 5, 4 or 3. Usually, two base chromosome numbers were present in each clade (Figure 2).

### 2.2. Chromosome Number Evolution

**Figure 3:**
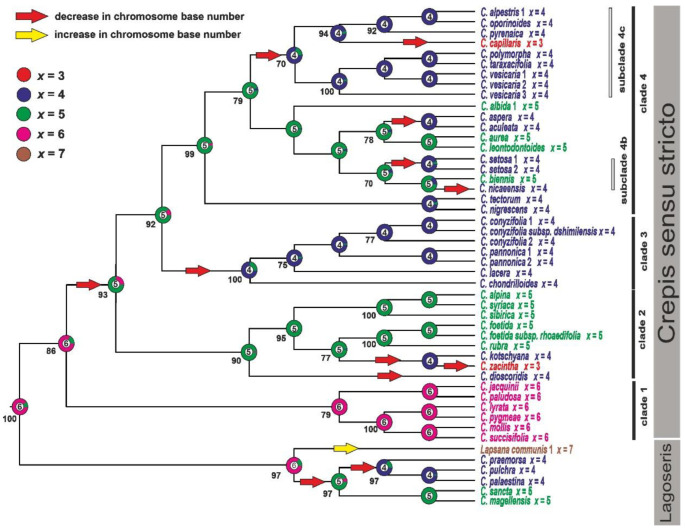
Ancestral character state reconstruction of the chromosome base numbers in Crepis s.l. The chromosome base numbers have been mapped on the ML tree resulting from analyses of the nrITS sequences using the maximum likelihood method as implemented in ChromEvol 2.0. The tree was rooted with *Picris hieracioides*, *Lactuca serriola* and *Sonchus oleraceus*. Bootstrap scores are shown below the branches.

Although the majority of the analysed Crepis species had base chromosome numbers of x = 5 (ten species) or x = 4 (21 species), some species were also found with x = 6 and x = 3. The x = 7 was only observed in *L. communis*. Most of the species were diploid, except for *C. vesicaria*, which had both diploid and tetraploid individuals. The ML analysis (ChromEvol 2.0), which was based on the nrITS datasets, enabled the ancestral chromosome base number for the common ancestor of Lagoseris and Crepis s.s. to be inferred as x = 6 (pp = 0.86). The analysis suggested 12 events of decreases (expectation above 0.5) in the chromosome base number, two in the Lagoseris lineage and ten in Crepis s.s.. Only one increase in the chromosome base number was inferred for *L. communis* in the *Lagoseris* lineage (Figure 3 and Table 1).

### 2.3. Chromosomal Organisation of the rDNA Loci

**Figure 4:**
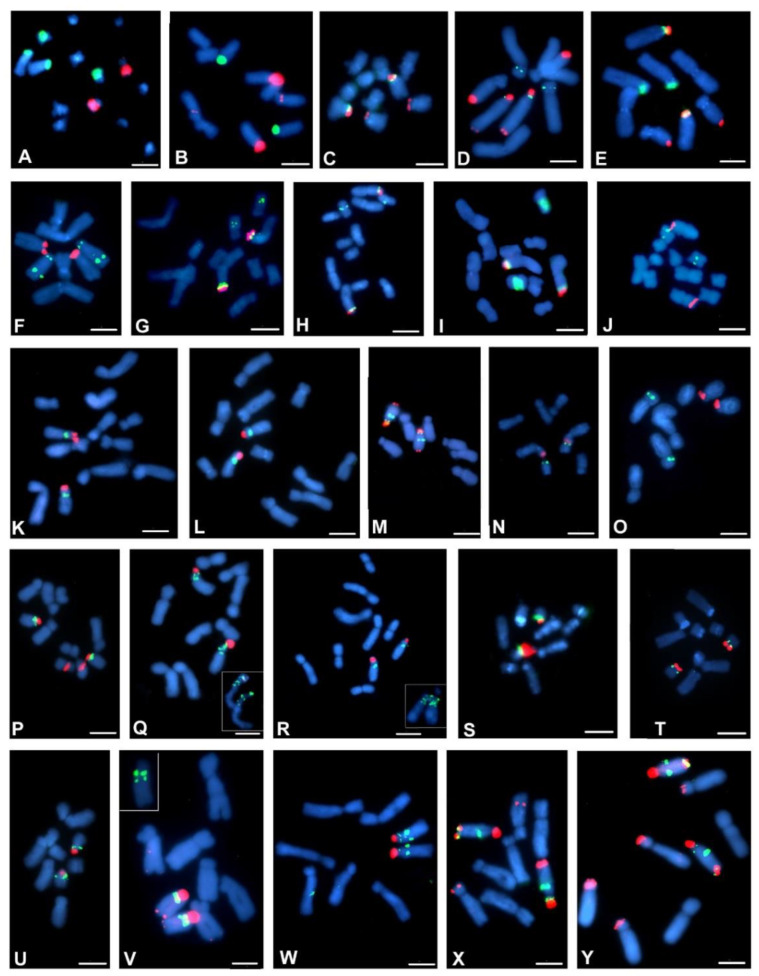
Localisation of 35S and 5S rDNA loci in metaphase chromosomes of Crepis species. Fluorescent in situ hybridisation was performed with the 5S rDNA probe (green fluorescence) and 25S rDNA probe (red fluorescence). (**A**) *Lapsana communis*, (**B**) *C. magellensis*, (**C**) *C. sancta*, (**D**) *C. palaestina*, (**E**) *C. pulchra*, (**F**) *C. praemorsa*, (**G**) *C. succisfolia*, (**H**) *C. mollis*, (**I**) *C. pygmeae*, (**J**) *C. lyrata*, (**K**) *C. paludosa*, (**L**) *C. jacquinii*, (**M**) *C. dioscoridis*, (**N**) *C. zacintha*, (**O**) *C. kotschyana*, (**P**) *C. rubra*, (**Q**) *C. foetida subsp. rhoaedifolia*, (**R**) *C. foetida*, (**S**) *C. sibirica*, (**T**) *C. syriaca*, (**U**) *C. alpina*, (**V**) *C. chondrilloides*, (**W**) *C. lacera*, (**X**) *C. pannonica* 1 and (**Y**) *C. pannonica* 2. Scale bar = 5 µm.

**Figure 5:**
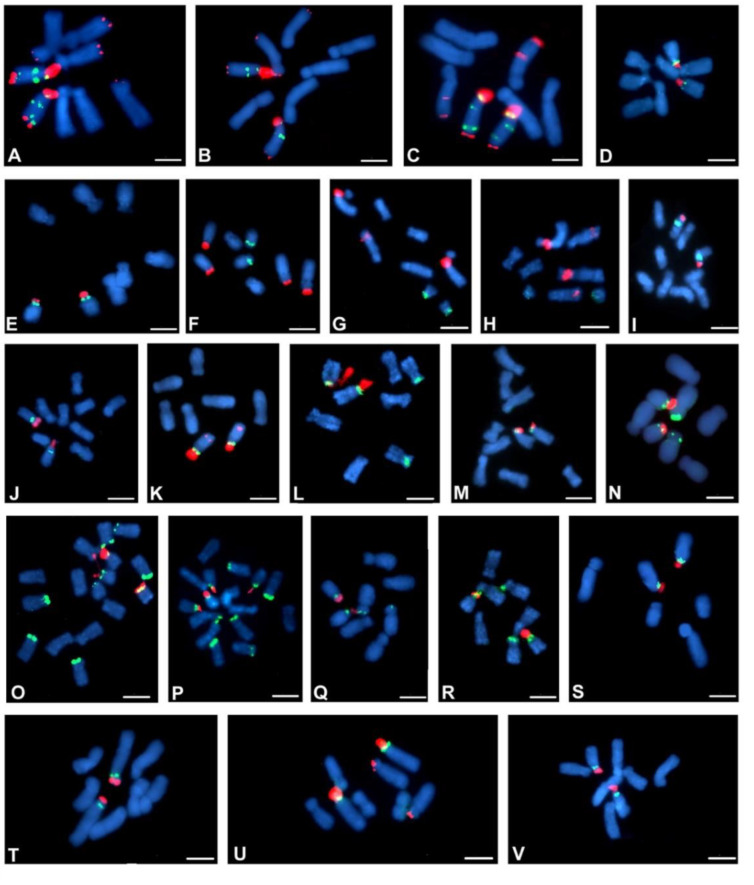
Localisation of 35S and 5S rDNA loci in metaphase chromosomes of Crepis species. Fluorescent in situ hybridisation was performed with 5S rDNA (green fluorescence) and 25S rDNA probes (red fluorescence). (**A**) *C. conyzifolia* 2, (**B**) C. *conyzifolia subsp. dshimilensis*, (**C**) *C. conyzifolia* 1, (**D**) *C. nigrescens*, (**E**) *C. tectorum*, (**F**) *C. nicaeensis*, (**G**) *C. setosa* 1, (**H**) *C. setosa* 2, (**I**) *C. leontodontoides*, (**J**) *C. aurea*, (**K**) *C. aculeata*, (**L**) *C. aspera*, (**M**) *C. albida*, (**N**) *C. vesicaria* 3, (**O**) *C. vesicaria* 2, (**P**) *C. vesicaria* 1, (**Q**) *C. taraxacifolia*, (**R**) *C. polymorpha*, (**S**) *C. capillaris*, (**T**) *C. pyrenaica*, (**U**) *C. oporinoides* and (**V**) *C. alpestris*. Scale bar = 5 µm.

The number and localisation of the rDNA loci was determined using FISH with 25S and 5S rDNA probes. The rDNA loci number and localisation are reported for 38 Crepis species and for *L. communis* for the first time (Table 1). Seventeen analysed diploid Crepis species had one locus of each 35S and 5S rDNA (Figure 4J–L,N–U and Figure 5D,E,I,J,L,M,S–V). Nine species had two loci of 35S rDNA (Figure 4B,C,E,P,U and Figure 5B,F,U,K), and only three species had three or four loci of this sequence (Figure 4D,X,Y and Figure 5A–C). Fourteen diploid species had two loci of 5S rDNA (Figure 4D–H,M,W,X and Figure 5B,L,N,Q,R). Three loci of 5S rDNA were observed in both accessions of *C. foetida* and in some of the accessions of *C. conyzifolia* and *C. pannonica* (Figure 4Q,R,Y and Figure 5A,C). *L. communis* (2n = 2x = 14) had three loci of 5S rDNA and one locus of 35S rDNA (Figure 4A; Table 1). The chromosomal organisation of the rDNA loci varied among the analysed species with more than 20 different patterns of the chromosomal distribution of the rDNA loci (Figure 4 and Figure 5 and Table 1). In the Lagoseris evolutionary lineage, there was high interspecific polymorphisms in the rDNA loci number and localisation. The number of both the 5S and 35S rDNA loci varied from one to three among these species, with each species having a unique chromosomal pattern of the rDNA loci distribution (Table 1 and Figure 4A–F).

In 12 species of the Crepis s.s. lineage, karyotypes with one chromosome bearing both rDNA loci within one, usually short, arm and with the locus of 35S rDNA in the more distal position, were observed (Table 1). Such a pattern was also observed in two species of clade 1 (*C. paludosa* and *C. jacquinii*; Figure 4K,L), three species in clade 2 (*C. sibirica*, *C. syriaca* and *C. alpina*; Figure 4S–U) and in eight species from clade 4 (Figure 5D,E,I,J,M,S,V,T). A similar pattern of both the rDNA loci in one chromosomal arm but with 5S rDNA in a more distal position (Figure 4N) was observed in *C. zacintha* karyotype (clade 2). In the karyotype of 16 species of Crepis s.s., in addition to the common pattern with both rDNA loci within one chromosome arm with the locus of 35S rDNA in a more distal position, additional locus/loci of 35S and/or 5S rDNA were observed (Figure 4G–J,M,P–S,V–Y and Figure 5A–C,K,L,N,Q,R,U and Table 1). Among them, the species from clade 3 had the relatively highest interspecific polymorphisms in the rDNA loci number and localisation.

Each species had a different pattern of rDNA loci distribution. Although quite variable loci patterns were observed in the species in clade 3, most of them had one chromosome that carried two loci of 35S rDNA and two or three loci of 5S rDNA (Figure 4V,X,Y and Figure 5A–C). In the karyotypes of *C. lyrata* (clade 1; Figure 4J) and three species from subclade 4b (*C. kotschyana*, *C. nicaeensis* and *C. setosa*; Figure 4O and Figure 5F–H), the 5S and 35S rDNA loci were observed in separate chromosomes. *C. vesicaria*, a species with both diploid and tetraploid accessions, had one chromosome bearing both the 35S and 5S rDNA in its short arm and the second locus of 5S rDNA in the short arm of another chromosome in the diploids (Figure 5N). The tetraploid accessions of *C. vesicaria* had the same patterns of loci distribution but with double the number of loci and chromosomes (Figure 5O,P).

**Figure 6:**
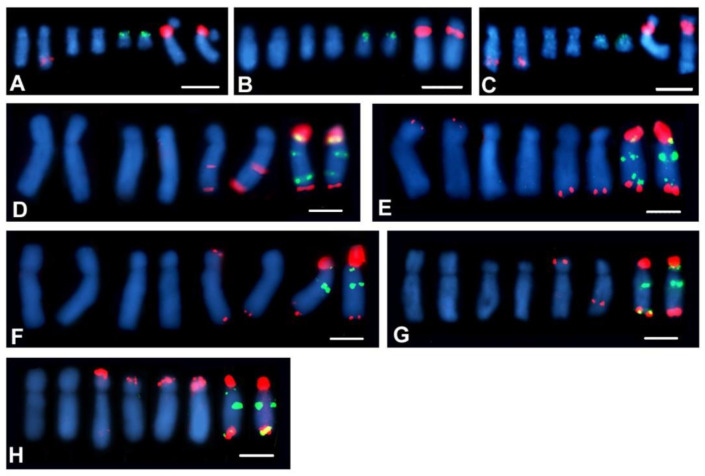
Karyotypes of Crepis species showing intraspecific polymorphisms of the rDNA loci number and localisation: (**A**) *C. setosa* 1, (**B**) *C. setosa* 2, (**C**) *C. setosa* 2, (**D**) *C. conyzifolia* 1, (**E**) *C. conyzifolia* 2, (**F**) *C. conyzifolia subsp. Dshimilensis*, (**G**) *C. pannonica* 1 and (**H**) *C. pannonica* 2. Scale bar = 5 µm.

Three diploid Crepis species had intraspecific polymorphisms of the number and/or localisation of the rDNA sites. A polymorphism of 35S rDNA loci number was observed among the analysed *C. setosa* accessions. Two, three or four hybridisation signals of 35S rDNA were observed (Figure 6A–C and Table 1). Among the analysed *C. conyzifolia* and *C. pannonica* accessions, there were polymorphisms of the chromosomal patterns of both the 35S and 5S rDNA loci (Figure 6D–F and Table 1) that had additional loci of both rDNA types.

### 2.4. Fluorochrome Banding

Fluorescent staining with CMA_3_ was used to detect the GC-rich chromosomal regions [33]. CMA_3_ banding was performed for 38 accessions representing 32 Crepis species and for *L. communis*. For the 28 Crepis species and *L. communis*, only the CMA_3_^+^ bands that co-localised with the major locus/loci of 35S rDNA were observed (Table 1 and Figure S4). In *C. aurea*, *C. dioscoridis* and *C. lacera* CMA_3_^+^, the bands colocalising with both the 35S rDNA and 5S rDNA loci were detected. In the *C. rubra* karyotype, four pairs of the CMA_3_^+^ bands were observed. Two of these were colocalised with the 35S rDNA loci, and two others did not colocalise with either the 35S or with the 5S rDNA loci.

### 2.5. Patterns of Ribosomal DNA Loci Evolution

**Figure 7:**
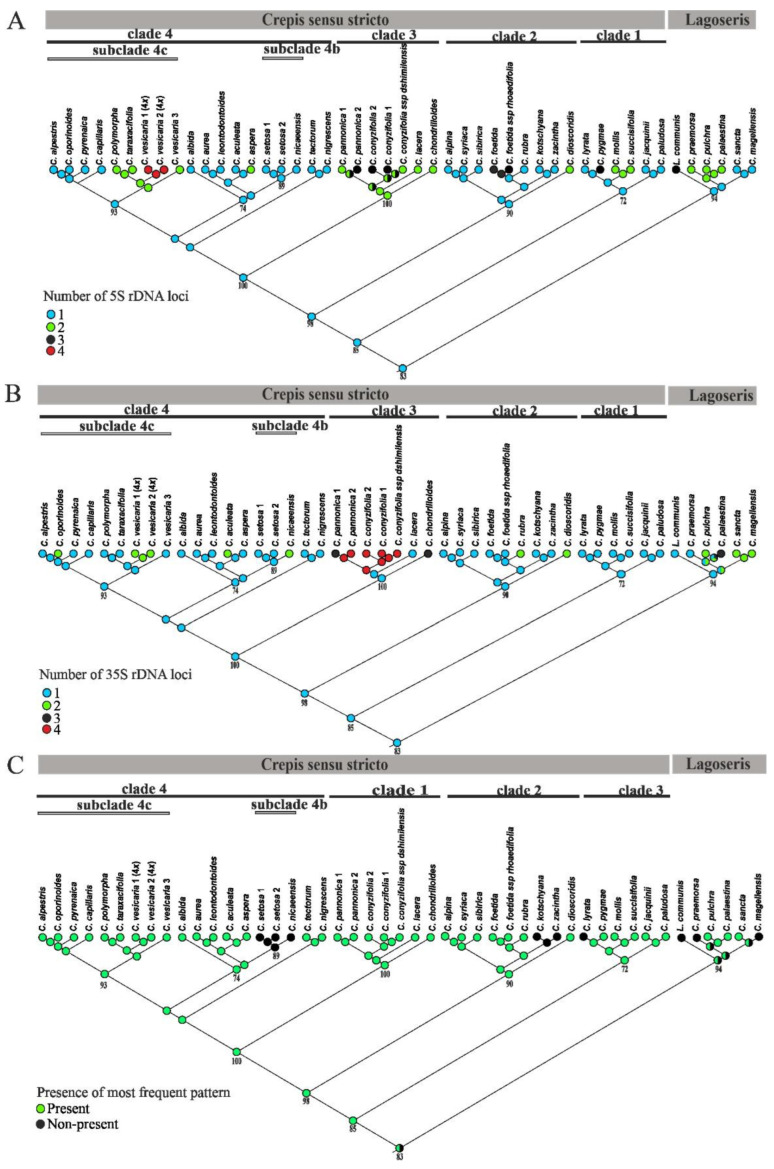
Ancestral character state reconstruction of the rDNA locus number and localisation for Crepis s.l. The numbers and localisation of the rDNA loci were mapped onto the ML tree of the nrITS sequences using maximum likelihood methods. (**A**) Number of 5S rDNA loci. (**B**) Number of 35S rDNA. (**C**) Presence/absence of the chromosome carrying both 35S and 5S rDNA loci within the same arm.

The number of rDNA loci was mapped on the ML phylogenetic tree of the nrITS using the maximum likelihood reconstruction methods as implemented in Mesquite (Figure 7). One locus of 5S rDNA was reconstructed as an ancestral state for the Lagoseris and Crepis s.s. lineages (Figure 7A). In the Lagoseris clade, only two species (*C. sancta* and *C. magellensis*) had an ancestral number of loci. The remaining species had two or three loci of 5S rDNA. In the Crepis s.s. lineage, more than half of the analysed species had an ancestral number of 5S rDNA loci. Thirteen events of 5S rDNA loci gains were reconstructed for this evolutionary lineage. Gains of the 5S rDNA loci were inferred for a common ancestor of clade 3 and a common ancestor of the species related to *C. vesicaria* (clade 4). Other gains of 5S rDNA were reconstructed at the tips of the tree (Figure 7A).

Analyses of the 35S rDNA loci numbers resulted in the reconstruction of one locus of 35S rDNA as an ancestral state for a common ancestor of Lagoseris and Crepis s.s. (Figure 7B). Most species of the Lagoseris lineage had a higher number of 35S rDNA loci (two or three loci), and only two species, *C. praemorsa* and *L. communis*, had only one locus (Figure 7B). Conversely, most species of the Crepis s.s. lineage had an ancestral number of 35S rDNA loci, except for clade, 3 in which the majority of species had three or four loci. All of the species in clade 1 and most of the species in clade 2 and clade 4 had an ancestral loci number (Figure 7B). Nine events of 35S rDNA loci gains that had accompanied the speciation or evolution of two closely related species were reconstructed in Crepis s.s. (Figure 7B).

In the karyotypes of the majority of the analysed species, one chromosome consistently carried one of each 35S and 5S rDNA loci in the same, often short, chromosomal arm with 35S rDNA located distally. The presence/absence of this chromosome was mapped on the ML phylogenetic tree of the nrITS using the maximum likelihood reconstruction. The ancestral state that was reconstructed for the Lagoseris lineages was ambiguous (Figure 7C). The analysis resulted in the reconstruction of this arrangement as an ancestral state for Crepis s.s. (Figure 7C). Only three events of rDNA loci repositioning were reconstructed for Crepis s.s. During the evolution of a common ancestor of subclade 4b and the speciation of *C. lyrata* and *C. kotschyana*, the repositioning of the rDNA loci resulted in karyograms with the 5S and 35S rDNA loci in different chromosomes. However, the result of the rDNA loci repositioning during the speciation of *C. zacintha* was a karyotype with both rDNA locus types in one chromosomal arm but in the reverse order of the 5S and 35S rDNA loci (Figure 4N and Table 1).

## 3. Discussion

Understanding the phylogenetic relationships between organisms is a prerequisite for almost any evolutionary study. Previous analyses of Crepis phylogeny revealed incongruences between the plastid and nrITS-based phylogenies [26,27]. The phylogenetic relationships that were inferred from the 5S rDNA NTS analyses were largely congruent with the nrITS-based phylogeny for the Lagoseris lineage but less so for the Crepis s.s. species. In the latter lineage, only a few basal nodes had good support. Nevertheless, the groups of closely related species that were recovered from analyses of both nuclear rDNA markers were largely congruent with previous studies [26,27]. The species with x = 6 (Table 1) that were monophyletic in the nrITS phylogeny were recovered in three well-supported clades (clades A, B and C) in the 5S rDNA NTS analysis.

Well-supported clade D, which primarily comprises perennial species that are related to the *C. conyzifolia* recovered in clade 3 in the ITS-based phylogeny, additionally included the annual *C. dioscoridis* in the 5S rDNA NTS-based phylogeny, which was similar to the cpDNA phylogeny [27]. This close relationship of *C. dioscoridis* to other species of clade D was also supported by cytogenetic analyses of the karyotype formula and rDNA loci distribution. Clades F and J corresponded to clade 2 in their ITS phylogeny, whereas clades E G, H, I and K corresponded to clade 4.

Unlike nrITS, which often showed genomic uniformity, 5S rDNA NTS sequences were usually highly polymorphic within individuals due to the absence of interlocus homogenisation [34]. The 5S rDNA NTS of different arrays had the potential to evolve independently. This may have led to multiple gene families in some diploid, as well as polyploid, plant species [35,36,37]. Despite this limitation, 5S rDNA NTS has been found to be very informative both at the intergeneric and at the species levels in many taxonomical groups (e.g., Refs. [38,39,40]), but there are also genera where this marker does not yield high resolution at either level [41]. Most of the species had only one dominant type of the 5S rDNA NTS variant, which is similar, as has previously been shown for many diploid species [42], although some intraindividual variations in the NTS region were recovered in some species. More than one type of 5S rDNA NTS were observed in some species that had more than one locus of 5S rDNA (Figure 1 and Figure 2). Several reasons for such heterogeneity of 5S rDNA in diploids have been proposed, including an inefficient interlocus recombination that leads to a poor homogenisation or introgression [42,43].

The current study provided novel data on the 5S and 35S rDNA loci localisation for 38 species of Crepis and *L. communis*. Most of the analysed species had one locus of 35S rDNA in a subterminal chromosomal position and one interstitial 5S rDNA locus, which is an arrangement that is typical for many eudicots [44], including some taxa of the Asteraceae family, e.g., Lactuca [45], or species from the tribe Hieraciinae [34]. A quite characteristic feature of the karyotypes of most Crepis species was the presence of one chromosome that carried one of each 35S and 5S rDNA in one, usually short arm, with a 35S rDNA locus in the more distal and the 5S rDNA locus in the more proximal positions relative to one another. Although such an arrangement of rDNA loci is relatively rare among angiosperm species that have a single locus of each rDNA [44,46], in Crepis, this pattern has often been observed in karyotypes with either a single locus or multiple loci of rDNAs. Moreover, earlier reports on Crepis species revealed a similar arrangement of rDNA [30,31,32].

**Figure 8:**
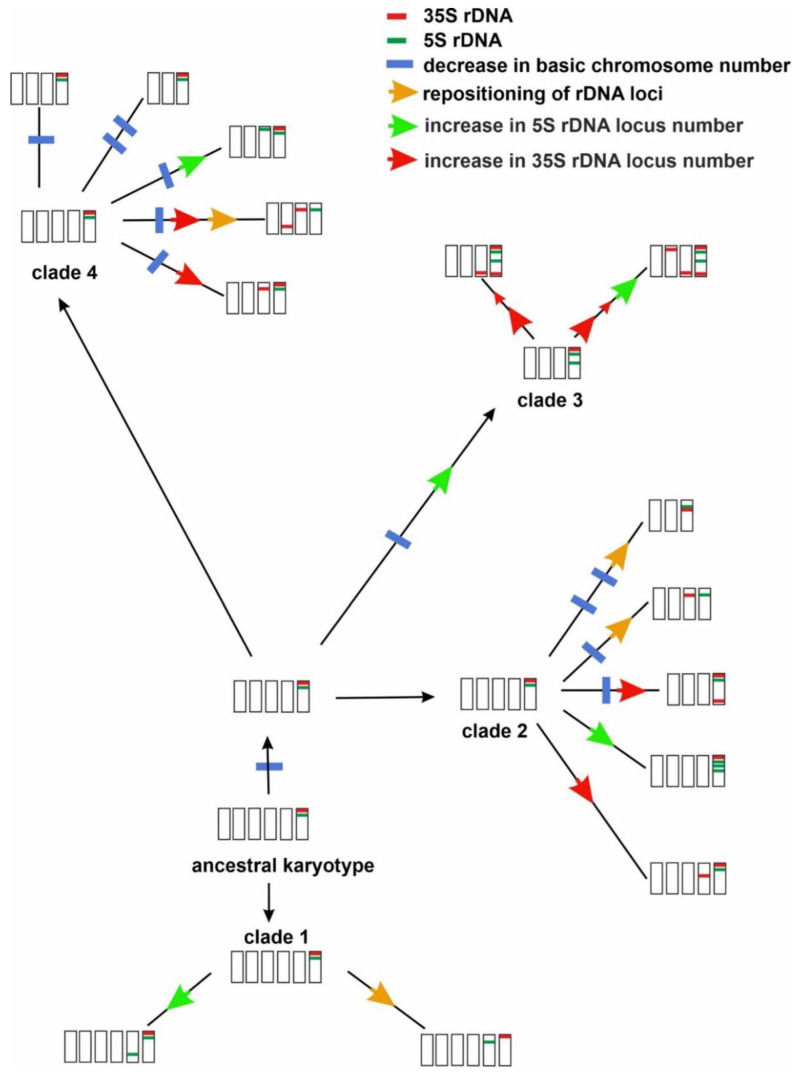
Hypothesis on the evolution of base chromosome numbers and rDNA loci number and localisation in Crepis s.s.

The interpretation of the rDNA loci distribution patterns in a phylogenetic context not only enables a better understanding of their evolution but also provides an insight into the evolution of the whole karyotype structure [21,22,23]. The most common reconstructed ancestral character state was the presence of a chromosome with both rDNA loci in one arm in the Crepis karyotype. Generally, the ancestral state of the rDNA loci number for Crepis s.l., but, also, for both lineages Lagoseris and Crepis s.s. was reconstructed as one locus of each 35S and 5S rDNA (Figure 8 and Supplementary Figure S4). Most of the analysed Crepis species had a larger number of rDNA loci, and relatively high interspecific polymorphisms in rDNA loci chromosomal organisation were observed. High variability in the number and localisation of rDNA loci were reported in many different plant genera, and usually, a higher polymorphism of the 35S rDNA locus number and distribution is common in plants [47,48,49]. The numbers of the increases in the 5S and 35S rDNA loci were quite similar in the analysed Crepis species, and the observed polymorphisms in the number and localisation of both rDNA loci were also comparable. The differences in the number and localisation of rDNA loci in related species have been assigned to various mechanisms, e.g., chromosomal rearrangements such as locus duplication/deletion and transposon-mediated transposition events [50,51,52,53,54]. The coding regions of rDNA sequences are an evolutionary very conserved fraction in the eucaryotic genome. This conservatism, however, appears to be a powerful source for genome instability, because the chromosomes that carry rDNA arrays (especially in subtelomeric regions) may be subject to unequal recombination and recombination between nonhomologous loci [55,56].

The ancestral state of the presence/absence of the chromosome carrying both rDNA locus types in one chromosome arm could not be unambiguously inferred for the Lagoseris lineage. Two or three events of rDNA loci repositioning explained their distribution patterns in extant species of this lineage. The repositioning of the rDNA loci has usually not been inferred for the same branches for which the changes in the base chromosome numbers in Lagoseris were inferred (Figure S5 [27]).

The common ancestor of the Crepis s.s. lineage was inferred to have had x = 6 and one locus of each of 35S and 5S rDNA in the same chromosomal arm with the 35S rDNA locus in a more distal position (Figure 8 and Figure S5).

Most species of clade 1 had such a chromosome in their karyotype, and only one event of rDNA loci repositioning was inferred (the locus of the 35S rDNA and 5S rDNA in separate chromosomes). 5S rDNA loci gains were inferred for the evolution of three species in this clade. The evolution of the common ancestor of the species from clades 2, 3 and 4 accompanied a descending dysploidy (from x = 6 to x = 5). Further changes in the base chromosome number (from x = 5 to x = 4 and from x = 4 to x = 3), as well as both repositioning and gains of the rDNA loci, were reconstructed for the diversification and speciation of the taxa from clade 2 (Figure 8). The loci repositioning and descending dysploidy were supported in the same lineages, whereas the gains in the 35S rDNA loci numbers were inferred in lineages with a base chromosome number of x = 5 or x = 4, and the gain of 5S rDNA locus was inferred for line with x = 5 (Figure 8 and Figure S5). The evolution of the common ancestor of clade 3 was accompanied by descending dysploidy and a gain of the 5S rDNA loci. Recently published data on genome size evolution in Crepis has also revealed a relatively large genome size increase during the evolution of this clade [27] that encompasses species with x = 4 exclusively. The further diversification in this clade was accompanied by a few events of rDNA loci gains and, as was earlier shown [27], increases in the genome size. However, it should be emphasised that the increases in genome size are primarily due to retrotransposons amplification in the plant [57], and the strong correlation between the increase in the number of rDNA loci and increases in genome size has not been described in plants [22,58,59]. Two species from this clade revealed relatively high intraspecific polymorphisms in rDNA loci chromosomal distribution. Silva et al. [60] showed that the amplification of repetitive sequences, mostly LTR retrotransposons, may shape the karyotype structure, promoting chromosome rearranging and changes in the chromosomal distribution of tandem repeats.

Clade 4, which is the most species-rich group of Crepis s.s., consists of species with base chromosome numbers of x = 5, 4 and 3. Its ancestral karyotype was reconstructed as having x = 5 and one locus of each 35S and 5S rDNA within the same chromosomal arm. Five events of subsequent descending dysploidy were inferred for this clade. One of these was reconstructed for the same lineage in which rDNA loci repositioning has occurred (subclade 4c). Three events of 5S rDNA loci gains and three events of 35S rDNA loci gains were inferred for this clade albeit in different lineages. Some events of descending dysploidy were inferred for the same lines in which increases of rDNA loci occurred (Figure 8 and Figure S5). rDNA loci chromosomal distribution was, in many taxa, used as a cytotaxonomic character that allows grouping phylogenetically related species [58,61,62]. On the contrary, most clades distinguished in Crepis s.l. contained species that showed various patterns of rDNA loci distribution, as well as chromosome number, karyotype formula and genome size [27], except for clade 3, which included only species with x = 5 and similar karyotype formula. A common tendency to increase genome size and rDNA loci number was also inferred for this clade [27]. High polymorphisms in rDNA loci distribution were observed also in Prospero, Chenopodium s.l., Brassica and many other genera [21,22,63].

The translocation of the 35S rDNA locus to a different chromosome could explain the variations that were observed in species that did not have the chromosome with both types of rDNA loci in the same chromosomal arm. However, other mechanisms such as transposon-mediated transposition or minor locus amplification/major locus reduction cannot be excluded [52,64,65]. Inversions, which often accompany karyotype evolution in plants [66], might have been involved in the rearrangement of the two types of rDNA loci that were observed in *C. zacintha* (Figure 8). The evolution of clade 3, which has quite variable patterns of rDNA loci distribution, is accompanied by increases in the genome size [27]. An increase in genome size has often been shown to be caused by the activation and/or amplification of the retrotransposons [67,68], which might also mediate rDNA loci repatterning [52,69,70]. However, other mechanisms such as the chromosomal rearrangements that are caused by unequal or ectopic recombination have also been postulated to play a role in karyotype rearrangements [51,52,64,65,71]. To gain a better understanding of the karyotype evolution in Crepis, comprehensive comparative analyses of repeatomes among Crepis species should be conducted. Such analyses not only will deliver new chromosomal markers but also give insight into mechanisms of genome size evolution in this taxon.

## 4. Materials and Methods

### 4.1. Plant Material and DNA Isolation

Forty-six accessions representing 39 Crepis species and *Lapsana communis* L. were used for the cytogenetic and molecular phylogenetic analyses. The plants were grown from seeds in a greenhouse facility of the University of Silesia under a 16 h/8 h photoperiod at 19 ± 2 °C. Vouchers were deposited at the Herbarium KTU (University of Silesia, Chorzów, Poland; Table 2). Total genomic DNA was isolated from fresh leaf tissue using the modified CTAB method [72]. Genomic DNAs were analysed for quality and quantity using a NanoDrop™ spectrophotometer (ND-1000, peqLab, Erlangen, Germany). The 5S rDNA NTS sequences were amplified from the same DNA extracts as the nrITS that were reported by Senderowicz et al. [27]. The FISH results were obtained for five to ten individuals per accession, except for *C. chondrilloides* (two individuals) and *C. nigrescens* (three individuals), and always included the individuals that had been used for the DNA extraction.

### 4.2. DNA Amplification and Sequencing

The internal transcribed spacers of the 35S rRNA gene (nrITS, including ITS1, the intervening 5.8S rDNA and ITS2) were amplified from the *C. chondrilloides* genome using a primer pair anchored in 18S rDNA and 25S rDNA (18S dir [5′-CGTAACAAGGTTTCCGTAGG-3′] and 25S com [5′-AGCGGGTAGTCCCGCCTGA-3′], as was published earlier [27,73]. The nucleotide sequence of nrITS isolated from *C. chondrilloides* is available in GenBank under number MZ226957. All of the other nrITS sequence data were obtained from GenBank (Supplementary Table S1).

The 5S rDNA non-transcribed spacer (5S rDNA NTS) region was amplified from the genomic DNAs that had been isolated from 45 accessions of Crepis, one accession of *Lapsana communis* and one accession of *Picris hieracioides*, which was used as an outgroup. The PCR amplification of the 5S rDNA NTS and the cloning of this region were performed according to Kolano et al. [74]. Positive colonies were transferred into clean Eppendorf tubes (1.5 mL), dissolved in 100 mL of ddH_2_O, incubated for 10 min at 96 °C and cooled down on ice for 10 min. These samples served as the DNA template for the PCR reactions using the M13 primers. The reactions were performed using Taq DNA Polymerase from *Thermus aquaticus* (SIGMA, St. Louis, MO, USA) according to the manufacturer’s instructions. The PCR amplification protocol consisted of an initial denaturation step of 2 min at 94 °C, followed by 35 cycles of amplification consisting of 40 s denaturation at 94 °C, 40 s of primer annealing at 55 °C and 40 s of DNA extension at 72 °C. All of the PCR products were treated with *E. coli* Exonuclease I and FastAP Termosensitive Alkaline Phosphatase (Thermo Fisher Scientific, Waltham, MA, USA) according to the manufacturer’s instructions. The product sequencing was performed by Macrogen (Amsterdam, The Netherlands) using a 3730xl DNA Analyser (Applied Biosystems, Foster City, CA, USA). At least five clones were analysed for each diploid accession and ten clones for each tetraploid accession. All of the sequences were deposited in GenBank (the accession numbers are listed in Table 2).

### 4.3. Phylogenetic Analyses, Inferences of the Patterns of the Evolution of the rDNA Loci and Ancestral State Reconstructions

Phylogenetic analyses were performed using the DNA sequences of 5S rDNA NTS and the nrITS regions obtained in this study and those published earlier (Supplementary Table S1 [27]), which represent multiple species of two evolutionary lineages of the genus Lagoseris and Crepis s.s. Multiple sequence alignments for both datasets were performed 20 times using webPRANK [75] and MergeAlign [76] in order to obtain a consensus multiple sequence alignment. The phylogenetic relationships were inferred using maximum likelihood (ML) analyses as implemented in IQ-TREE version 0.9.5 [77]. *Picris hieracioides*, *Lactuca seriola* and *Sonchus oleraceus* were used as the outgroup taxa. The ITS dataset comprised all of the studied species. The 5S rDNA NTS sequences were too variable to obtain a reliable alignment in the phylogenetic analyses. Therefore, the analyses were performed separately for the Lagoseris and Crepis s.s. evolutionary lineages. The significance of the inferred relationships was assessed via bootstrapping with 1000 replicates. The most appropriate model of sequence evolution for the ML analyses was determined using the Bayesian information criterion as implemented in IQ-TREE. The best-fit model that was selected for the nrITS was TIM3e + G4. For analyses of the 5S rDNA NTS, the best-fit models were K2P + G4 for *Lagoseris* (rooted with *Picris hieracioides*) and K3P + G4 for Crepis s.s. lineage (rooted with *C. praemorsa*).

The phylogram that resulted from the ML analysis was used to infer the evolution of the chromosomal organisation of the rDNA loci and base chromosome number. The analyses were performed using the better-supported nrITS phylogram. Three characters were analysed separately: (i) the number of 5S rDNA loci, (ii) the number of 35S rDNA loci and (iii) the presence/absence of the chromosome carrying both the 5S and 35S rDNA loci within the same arm. The analysis was performed using the ML method as implemented in Mesquite [78]. The nrITS phylogram was also used to infer the evolution of the base chromosome numbers with ChromEvol. The maximum likelihood analysis was performed under the CONST_RATE model as implemented in ChromEvol 2.0. software [79]. For the ChromEvol analyses, the best-fit model was tested using an AIC test (Table S2). For the best-fitted model, the analyses were rerun with parameters that were fixed to those that were optimised in the first run using 10,000 simulations to compute the expected number of changes along each branch, as well as the ancestral haploid chromosome numbers at the nodes.

### 4.4. Chromosome Preparation and Fluorescent In Situ Hybridisation

Young leaves were used as the material to prepare the chromosomal spreads. The leaves were pre-treated with 2-mM 8-hydroxyquinoline for 2 h at room temperature and 2 h at 4 °C, fixed in methanol:glacial acetic (3:1) and stored at −20 °C until they were used. The mitotic metaphase chromosome spreads were prepared according to Dydak et al. [80] using an enzyme mixture consisting of 20% pectinase (SIGMA, St. Louis, MO, USA) and 4% cellulose (Onozuka R-10; Serva, Heidelberg, Germany). The coding region of the 25S rRNA that was isolated from *Arabidopsis thaliana* [81] and labelled with rhodamine-4-dUTP (Roche, Basel, Switzerland) was used to detect the 35S rDNA loci. The 5S rDNA monomer that was isolated from *Triticum aestivum* [82] and labelled with digoxigenin-11-dUTP (Roche, Basel, Switzerland) was used to detect the 5S rDNA loci. Both probes were labelled using nick translation according to manufacturer’s instructions (Roche, Basel, Switzerland). FISH was performed according to Kolano et al. [74]. The hybridisation mixture consisting of 100 ng of each labelled DNA probe, 50% formamide, 2 × SSC and 10% dextran sulphate was denatured for 10 min at 95 °C and immediately cooled down on ice. The denaturation of the slides and the hybridisation mixture were performed on an Omnislide Thermal cycler (ThermoHybaid, Franklin, MA, USA) at 72 °C for 4 min. Hybridisation was conducted for 48 h at 37 °C in a humid chamber. Stringent washes (0.1 × SSC at 42 °C) were followed by the detection of digoxigenin using the FITC-conjugated primary anti-digoxigenin antibody (Roche, Basel, Switzerland). The signals were then amplified using secondary FITC-conjugated anti-sheep antibodies (Jackson ImmunoResearch, West Grove, PA, USA). The slides were analysed under a Zeiss AxioImager.Z.2 fluorescence microscope (Zeiss, Aalen, Germany); the images were acquired with an AxioCam HMr camera attached to an AxioImager.Z.2 wide-field epifluorescence microscope (both Zeiss, Oberkochen, Germany) and processed uniformly using ZEN 2.3 Pro (Zeiss) and Photoshop CS3 (Adobe, San Jose, CA, USA).

### 4.5. Fluorochrome Banding

The chromosomal spreads that were used for CMA3 banding were prepared according to Dydak et al. [80]. Double-fluorescence staining with chromomycin A_3_ (CMA_3_) and 4′,6-diamidino-2-phenylindole (DAPI) was performed as described by Kolano et al. [83]. The slides were analysed with an Olympus PROVIS AX70 fluorescence microscope (Olympus, Tokyo, Japan), and the images were acquired with a Retiga-2000R Fast1394 camera (QImaging, Surrey, BC, Canada).

## 5. Conclusions

The patterns of the number and localisation of the 5S and 35S rDNA loci in Crepis species revealed an interspecific variation. Some of these characters were shared by most of the analysed species, notably the presence of a chromosome that carried both the 35S and 5S rDNA loci within the same arm. A hypothetical ancestral karyotype of Crepis s.l. was reconstructed as having one locus of each 5S and 35S rDNA. The common ancestor of Crepis s.s. carried these two rDNA locus types in one chromosomal arm. Several events of both 35S and 5S rDNA loci repatterning, which involved the repositioning of the loci and a change of their numbers, were inferred to have accompanied the evolution of the rDNA loci. Some of the changes in rDNA loci repatterning seem to coincide with descending dysploidy. This study provides the first comprehensive in-depth analysis of the karyotypes of numerous Crepis species beyond their chromosome numbers and karyotype structures. It should serve as the basis for more detailed analyses of the Crepis genomes using more chromosomal markers that represent various repetitive DNA families.

## Figures and Tables

**Table 1:** The number and localisation of the 35S and 5S rDNA loci and CMA_3_^+^ bands in Crepis.

Species	Chromosome Number	Number and Localisation of 35S 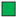 and 5S 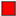 rDNA Loci and CMA_3_^+^ 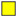 Bands			
		5S rDNA	35S rDNA		CMA_3_^+^
**Crepis s.s.**					
*Crepis aculeata*	2n = 2x = 8	1	2	^ 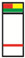 ^	1 (35S rDNA) *
*C. albida*	2n = 2x = 10	1	1	^ 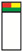 ^	1 (35S rDNA)
*C. alpestris*	2n = 2x = 8	1	1	^ 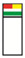 ^	1 (35S rDNA)
*C. alpina*	2n = 2x = 10	1	1	^ 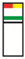 ^	1 (35S rDNA)
*C. aspera*	2n = 2x = 8	2	1	^ 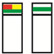 ^	-
*C. aurea*	2n = 2x = 10	1	1	^ 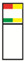 ^	2 (1 with 35S & 1 with 5S rDNA)
*C. capillaris*	2n = 2x = 6	1	1	^ 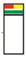 ^	1 (35S rDNA)
*C. conyzifolia* (1)	2n = 2x = 8	3	4	^ 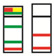 ^	1 (35S rDNA)
*C. conyzifolia* (2)	2n = 2x = 8	3	4	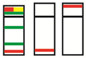	1 (35S rDNA)
*C. conyzifolia subsp. dshimilensis* (3)	2n = 2x = 8	2	4	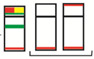	1 (35S rDNA)
*C. chondrilloides*	2n = 2x = 8	1	3	^ 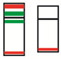 ^	-
*C. dioscoridis*	2n = 2x = 8	2	2	^ 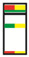 ^	3 (2 with 35S & 1 with 5S rDNA)
*C. foetida* (1)	2n = 2x = 10	1	1	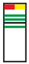	1 (35S rDNA)
*C. foetida subsp. rhoaedifolia* (2)	2n = 2x = 10	3	1	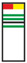	1 (35S rDNA)
*C. jacquinii*	2n = 2x = 12	1	1	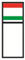	-
*C. kotschyana*	2n = 2x = 8	1	1	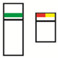	1 (35S rDNA)
*C. lacera*	2n = 2x = 8	2	1	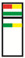	2 (1 with 35S & 1 with 5S rDNA)
*C. leontodontoides*	2n = 2x = 10	1	1	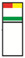	1 (35S rDNA)
*C. lyrata*	2n = 2x = 12	1	1	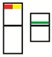	1 (35S rDNA)
*C. mollis*	2n = 2x = 12	2	1	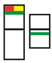	1 (35S rDNA)
*C. nicaeensis*	2n = 2x = 8	1	2	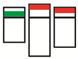	-
*C. nigrescens*	2n = 2x = 8	1	1	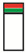	-
*C. oporinoides*	2n = 2x = 8	1	2	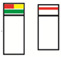	1 (35S rDNA)
*C. paludosa*	2n = 2x = 12	1	1	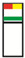	1 (35S rDNA)
*C. pannonica* (1)	2n = 2x = 8	2	3	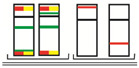	2 (35S rDNA)
*C. pannonica* (2)	2n = 2x = 8	3	4	^ 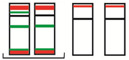 ^	-
*C. polymorpha*	2n = 2x = 8	2	1	^ 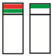 ^	-
*C. pygmeae*	2n = 2x = 12	3	1	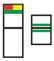	1 (35S rDNA)
*C. pyrenaica*	2n = 2x = 8	1	1	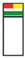	1 (35S rDNA)
*C. rubra*	2n = 2x = 10	1	2	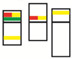	4 (2 with 35S rDNA)
*C. setosa* 1	2n = 2x = 8	1	1 + (1)	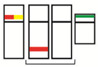	1 (35S rDNA)
*C. setosa* 2	2n = 2x = 8	1	1 or 2	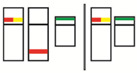	1 (35S rDNA)
*C. sibirica*	2n = 2x = 10	1	1	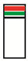	-
*C. succisifolia*	2n = 2x = 12	2	1	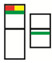	1 (35S rDNA)
*C. syriaca*	2n = 2x = 10	1	1	^ 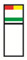 ^	1 (35S rDNA)
*C. taraxacifolia*	2n = 2x = 8	2	1	^ 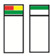 ^	1 (35S rDNA)
*C. tectorum*	2n = 2x = 8	1	1	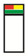	1 (35S rDNA)
*C. vesicaria* (3)	2n = 2x = 8	2	1	^ 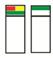 ^	1 (35S rDNA)
*C. vesicaria* (1)	2n = 4x = 16	2	1	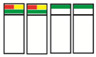	2 (35S rDNA)
*C. vesicaria* (2)	2n = 4x = 16	2	1	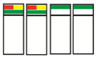	2 (35S rDNA)
*C. zacintha*	2n = 2x = 6	1	1	^ 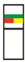 ^	1 (35S rDNA)
**Lagoseris**					
*C. magellensis*	2n = 2x = 10	1	2	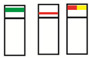	1 (35S rDNA)
*C. palaestina*	2n = 2x = 8	2	3	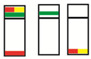	2 (35S rDNA)
*C. praemorsa*	2n = 2x = 8	2	1	^ 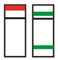 ^	-
*C. pulchra*	2n = 2x = 8	2	2	^ 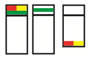 ^	2 (35S rDNA)
*C. sancta*	2n = 2x = 10	1	2	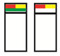	2 (35S rDNA)
*Lapsana communis*	2n = 2x = 14	3	1	^ 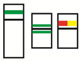 ^	1 (35S rDNA)

* The co-localisation of the CMA_3_^+^ bands with the rDNA loci are given in parentheses. A parenthesis under the chromosome in the idiograms indicates a polymorphism in the rDNA loci chromosomal organisation.

**Table 2:** Species name, collection number and voucher number of the analysed taxa and GenBank accession numbers of the sequences obtained in this research.

Species	Collection Number	Voucher Number	5S rDNA NTS
			GenBank Number
**Crepis s.s.**			
*Crepis aculeata* Boiss.	BGT 38	KTU154623	MZ226690–MZ226694
*C. albida* Vill.	UGA 233	-	MZ226725–MZ226729
*C. alpestris* (Jacq.) Tausch	BGUG N49	KTU157712	MZ226695–MZ226699
*C. alpina* L.	USDA PI 274367	KTU154609	MZ226705–MZ226709
*C. aspera* L.	LBG 006722	KTU157716	MZ226715–MZ226719
*C. aurea* (L.) Cass.	USDA PI 312843	KTU157719	MZ226720–MZ226724
*C. capillaris* Wallr.	BGGU 335	KTU154610	MZ226735–MZ226739
*C. conyzifolia* (Gouan) A.Kern. (1)	GBA 462	KTU157720	MZ226740–MZ226744
*C. conyzifolia* (Gouan) A.Kern. (2)	UGA 236	-	MZ226745–MZ226749
*C. conyzifolia subsp. dshimilensis* (K.Koch) Lamond (3)	GBBG	-	MZ226839–MZ226843
*C. chondrilloides* Jacq.	Triest, Italy 45°36′46.54″ N 13°51′36.96″ E	-	MZ226952–MZ226956
*C. dioscoridis* L.	IPK CRE2	KTU154619	MZ226750–MZ226754
*C. foetida* L.	USDA PI 296071	KTU154612	MZ226755–MZ226759
*C. foetida subsp. rhoaedifolia* (M.Bieb.) Celak.	HBBH 1734	KTU154614	MZ226864–MZ226868
*C. jacquinii* Tausch	Sarnia Skała Tatra Mts, Poland 49°15′52.77″ N 19°56′30.36″ E	KTU159736	MZ226760–MZ226764
*C. kotschyana* Boiss.	USDA PI 310392	KTU164608	MZ226765–MZ226769
*C. lacera* Ten.	BGMN	KTU159735	MZ226770–MZ226774
*C. leontodontoides* All.	BGDG 658	KTU154631	MZ226775–MZ226779
*C. lyrata* (L.) Froel.	SSBG	-	MZ226780–MZ226785
*C. mollis* Asch.	Sławków, Poland 50°17′45.90″ N 19°16′59.06″ E	KTU154630	MZ226792–MZ226796
*C. nicaeensis* Balb.	BGEU	KTU157730	MZ226797–MZ226801
*C. nigrescens* Pohle	HUM	-	MZ226802–MZ226806
*C. oporinoides* Boiss. ex Froel.	ABGL 1516	KTU154622	MZ226710–MZ226714
*C. paludosa* Moench	Sławków, Poland 50°18′07.51″ N 19°21′19.10″ E	KTU154625	MZ226807–MZ226811
*C. pannonica* (Jacq.) K.Koch (1)	BGBD 256-01-00-14	KTU154627	MZ226817–MZ226826
*C. pannonica* (Jacq.) K.Koch (2)	BGEU	KTU157729	MZ226827–MZ226833
*C. polymorpha* Pourr	JBN 149	KTU157725	MZ226834–MZ226838
*C. pygmeae* L.	UGA 239	KTU157722	MZ226854–MZ226858
*C. pyrenaica* (L.) Greuter	BGBD 1010	KTU154621	MZ226859–MZ226863
*C. rubra* L.	BGK 364	KTU154607	MZ226869–MZ226873
*C. setosa* Haller f. 1	HBUR 1275	KTU154620	MZ226879–MZ226883
*C. sibirica* L.	BGBD 738	KTU157721	MZ226884–MZ226889
*C. succisifolia* Tausch	Rędziny, Poland 50°49′08.66″ N 15°55′55.27″ E	KTU154656	MZ226890–MZ226894
*C. syriaca* (Bornm.) Babc. & Navashin	KEW 0129064	KTU154615	MZ226895–MZ226899
*C. taraxacifolia* Thuill.	BGGU 347	KTU157723	MZ226900–MZ226904
*C. tectorum* L.	Ustroń, Poland 49°43′14.68″ N 18°49′29.11″ E	KTU157717	MZ226905–MZ226909
*C. veiscaria* L. 1 (4x)	BGBD 1014	KTU154616	MZ226910–MZ226920
*C. vesicaria* L. 2 (4x)	BGBD 918	KTU157726	MZ226921–MZ226931
*C. vesicaria* L. 3 (2x)	OBUP	KTU157724	MZ226932–MZ226936
*C. zacintha* (L.) Loisel.	BGT 92	KTU154606	MZ226937–MZ226941
**Lagoseris**			
*C. magellensis* F. Conti & Uzunov	BGMN	KTU157727	MZ226787–MZ226791
*C. palaestina* Bornm.	BGGU 335	KTU154611	MZ226812–MZ226816
*C. pulchra* L.	BGGU 341	KTU154648	MZ226849–MZ226853
*C. preamorsa* (L.) Tausch	BGBD 662	KTU154628	MZ226844–MZ226848
*C. sancta* (L.) Bornm.	BGUK 104	KTU154613	MZ226874–MZ226878
*Lapsana communis* L.	KEW 0018568	KTU154617	MZ226942–MZ226946
**Outgroup**			
*Picris hieracioides* L.	Jaworzno Poland 50°13′31.43″ N 19°16′28.63″ E	KTU157710	MZ226947–MZ226951

Voucher deposited in KTU; seed origin and accession number: (BGT) Botanic Garden of Tel Aviv University; (USDA) USDA North Central Regional Plant Introduction Station of the US National Plant Germplasm System; (UGA) Université Grenoble Alpes; (JBI) Jardín Botánico de Iturraran Lorategi Botanikoa, Spain; (BGUG) Botanical Garden of Universitat Graz; (BGBD) Botanical Garden Freie Universität Berlin—Dahlem; (LBG) Lyon Botanical Garden, France; (WB) Wołosate, Bieszczady National Park, Poland; (BGGU) The Botanical Garden of Göttingen University; (GBA) Giardino Botanico Alpino “Rezia”, Italy; (GBBG) Gruzja Batumi Botanical Garden; (IPK) The Leibniz Institute of Plant Genetics and Crop Plant Research (IPK), Germany; (HBBH) Hortus Botanicus Budapest, Hungary; (BGMN) Botanical Gardens of Majella National Park, Italy; (SSBG) The South-Siberian Botanical Garden of Altai State University; (BGEU) Botanical Garden of Eötvös University in Budapest; (HUM) Herbarium Univsersitatis Mosquensis; (ABGL) Alpine Botanical Garden of Lautaret, France; (JBN) Jardin Botanique de Nancy; (BGK) Botanical Garden in Kiel; (HBUR) Hortus Botanicus Universitatis, Romania; (KEW) Millenium Seed Bank KEW Gardens; (OBUP) Orto Botanico Dell Universito Di Padora Italia; (BGUK) Botanischer Garten Universität Konstanz, Germany.

## Data Availability

The nucleotide sequences are available in GenBank (http://www.ncbi.nlm.nih.gov/genbank, accessed on 28 February 2022) under numbers MZ226695–MZ226956 and MZ226957. Other data generated or analysed during this study are available from the corresponding author upon reasonable request.

## Supplementary Materials

The following supporting information can be downloaded at https://www.mdpi.com/article/10.3390/ijms23073643/s1.
