# Supplementary material for: Molecular and Cytogenetic Analysis of rDNA Evolution in Crepis Sensu Lato
Source: Int J Mol Sci. 2022 Mar 26;23(7):3643. doi: 10.3390/ijms23073643 (PMC8998684; doi:10.3390/ijms23073643)
Supplement: Supplementary file 1 [file ijms-23-03643-s001.zip › Senderowicz et al. Figure S5.pdf]

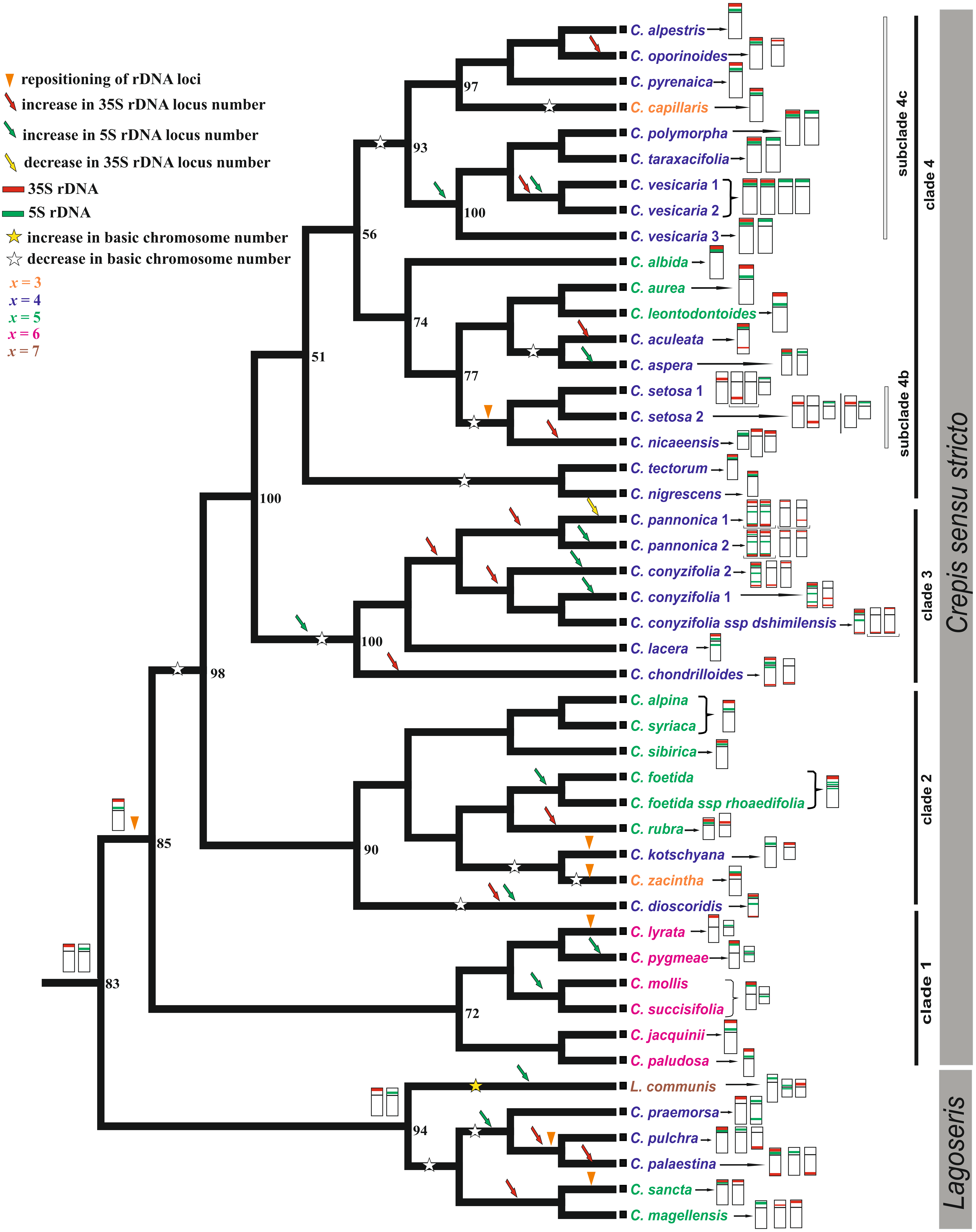

repositioning of rDNA loci
increase in 35S rDNA locus number
increase in 5S rDNA locus number
decrease in 35S rDNA locus number
35S rDNA
5S rDNA
increase in basic chromosome number
decrease in basic chromosome number
x = 3
x = 4
x = 5
x = 6
x = 7
C. alpestris
C. oporinoides
C. pyrenaica
C. capillaris
C. polymorpha
C. taraxacifolia
C. vesicaria 1
C. vesicaria 2
C. vesicaria 3
C. albida
C. aurea
C. leontodontoides
C. aculeata
C. aspera
C. setosa 1
C. setosa 2
C. nicaeensis
C. tectorum
C. nigrescens
C. pannonica 1
C. pannonica 2
C. conyzifolia 2
C. conyzifolia 1
C. conyzifolia ssp dshimilensis
C. lacera
C. chondrilloides
C. alpina
C. syriaca
C. sibirica
C. foetida
C. foetida ssp rhoeadifolia
C. rubra
C. kotschyana
C. zacintha
C. dioscoridis
C. lyrata
C. pygmeae
C. mollis
C. succisifolia
C. jacquinii
C. paludosa
L. communis
C. praemorsa
C. pulchra
C. palaestina
C. sancta
C. magellensis
97
93
100
56
74
77
51
100
100
98
90
85
72
83
94
subclade 4c
subclade 4b
clade 4
clade 3
clade 2
clade 1
Crepis sensu stricto
Lagoseris
