# Supplementary material for: Molecular and Cytogenetic Analysis of rDNA Evolution in Crepis Sensu Lato
Source: Int J Mol Sci. 2022 Mar 26;23(7):3643. doi: 10.3390/ijms23073643 (PMC8998684; doi:10.3390/ijms23073643)
Supplement: Supplementary file 1 [file ijms-23-03643-s001.zip › Senderowicz et al. Table S1.pdf]

Table S1. Species name and GenBank accessions numbers of the sequences used in this study

Species	GenBank accession number of nrITS
Crepis s.s.	
Crepis aculeata Boiss.	MN549102
C. albida (1) Vill.	MN549111
C. albida (2) Vill.	MN549112
C. alpestris (1) (Jacq.) Tausch	MN549104
C. alpestris (2) (Jacq.) Tausch	MN549105
C. alpina L.	MN549106
C. aspera L.	MN549108
C. aurea (L.) Cass.	MN549110
C. biennis L. 1	MN549113
C. biennis L. 2	MN549114
C. capillaris Wallr.	MN549116
C. conyzifolia (Gouan) A.Kern.	MN549117
C. conyzifolia (Gouan) A.Kern.	MN549118
C. conyzifolia subsp. Dshimilensis (K.Koch) Lamond	MN549132
C. dioscoridis L.	MN549119
C. foetida L.	MN549120
C. foetida subsp. rhoaedifolia (M.Bieb.) Celak.	MN549137
C. jacquinii Tausch	MT234671
C. kotschyana Boiss.	MN549122
C. lacera Ten.	MT234672

C. leontodontoides All.	MN549123
C. lyrata (L.) Froel.	MT234673
C. mollis Asch.	MN549125
C. nicaeensis Balb.	MT234675
C. nigrescens Pohle	MN549126
C. oporinoides Boiss. ex Froel.	MN549107
C. paludosa Moench	MN549128
C. pannonica (Jacq.) K.Koch 1	MN549130
C. pannonica (Jacq.) K.Koch 2	MT234676
C. polymorpha Pourr	MN549131
C. pontana Dalla Torre	MN549115
C. pygmae L.	MN549135
C. pyrenaica (L.) Greuter	MN549136
C. rubra L.	MN549138
C. setosa Haller f. 1	MN549140
C. setosa Haller f. 2	MN549141
C. sibirica L.	MN549142
C. succisifolia Tausch	MN549143
C. syriaca (Bornm.) Babc. & Navashin	MN549144
C. taraxacifolia Thuill.	MN549145
C. tectorum L.	MN549146
C. vesicaria L. 3	MN549149
C. vesicaria L. 2	MN549148
C. veiscaria L. 1	MN549147
C. zacintha (L.) Loisel.	MN549150

Lagoseris	
C. magellensis F.Conti & Uzunov	MT234674
C. palaestina Bornm.	MN549129
C. pulchra L.	MN549134
C. preamorsa (L.) Tausch	MN549133
C. sancta (L.) Bornm.	MN549139
Lapsana communis L. 1	MN549151
L. communis L. 2	MN549152
L. communis L. 3	MN549153
Outgroup	
Lactuca serriola L.	MN549156
Picris hieracioides L.	MN549154
Sonchus oleraceus L.	MN549157
