# Supplementary material for: Molecular and Cytogenetic Analysis of rDNA Evolution in Crepis Sensu Lato
Source: Int J Mol Sci. 2022 Mar 26;23(7):3643. doi: 10.3390/ijms23073643 (PMC8998684; doi:10.3390/ijms23073643)
Supplement: Supplementary file 1 [file ijms-23-03643-s001.zip › Senderowicz et al. Table S2.pdf]

Table S2

The ΔAIC scores and Akaike weights of each model tested in ChromEvol 2.0 software for nrITS data set.

Model*	Dataset	
	ITS	
	ΔAIC	Akaike weight
CR	**0**	**0.45**
CRD	0.2	0.40
CRDE	2	0.16
CRND	18.4	0.00
LR	108.3	0.00
LRD	108.3	0.00
LRDE	131.4	0.00
LRND	228	0.00

*CR – const_rate, CRD – const_rate_demi, CRDE – const_rate_demi_est, CRND – const_rate_no_dupl, LR – linear_rate, LRD – linear_rate_demi, LRDE – linear_rate_demi_est, LRND – linear_rate_no_dupl,
