# Supplementary figures and images for: Molecular and Cytogenetic Analysis of rDNA Evolution in Crepis Sensu Lato
Source: Int J Mol Sci. 2022 Mar 26;23(7):3643. doi: 10.3390/ijms23073643 (PMC8998684; doi:10.3390/ijms23073643)

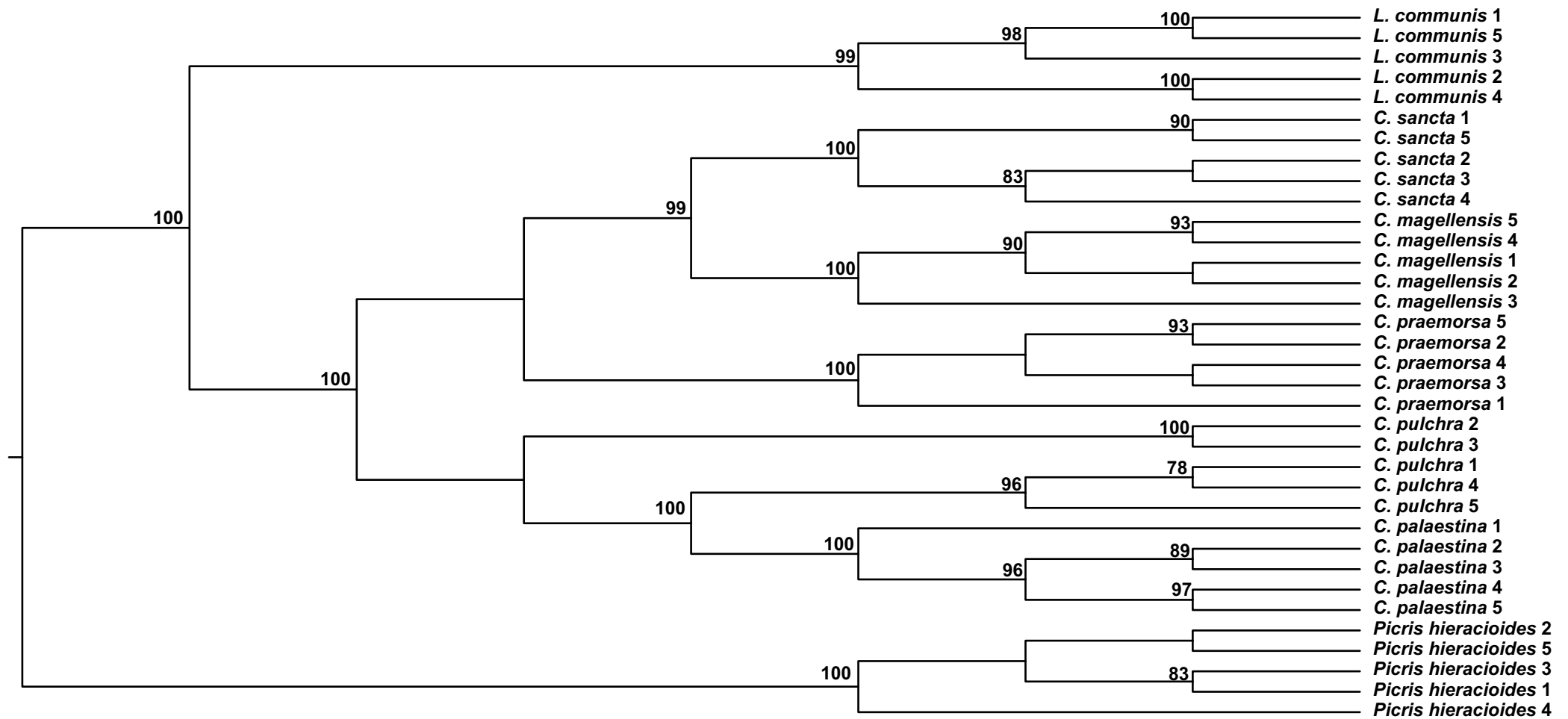

Supplement: Supplementary file 1 [file ijms-23-03643-s001.zip › Senderowicz et al. Figure S1.pdf]

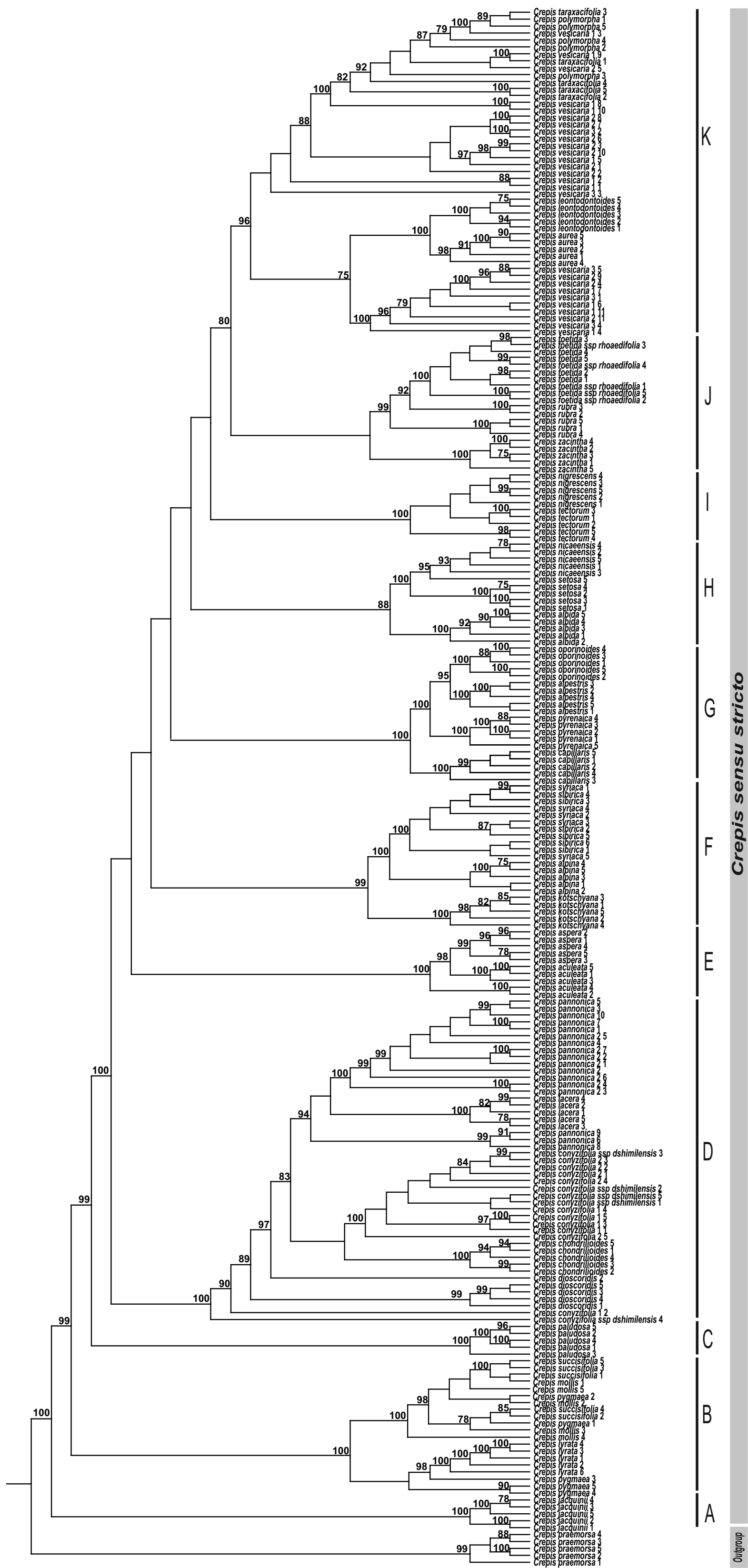

Supplement: Supplementary file 1 [file ijms-23-03643-s001.zip › Senderowicz et al. Figure S2.pdf]

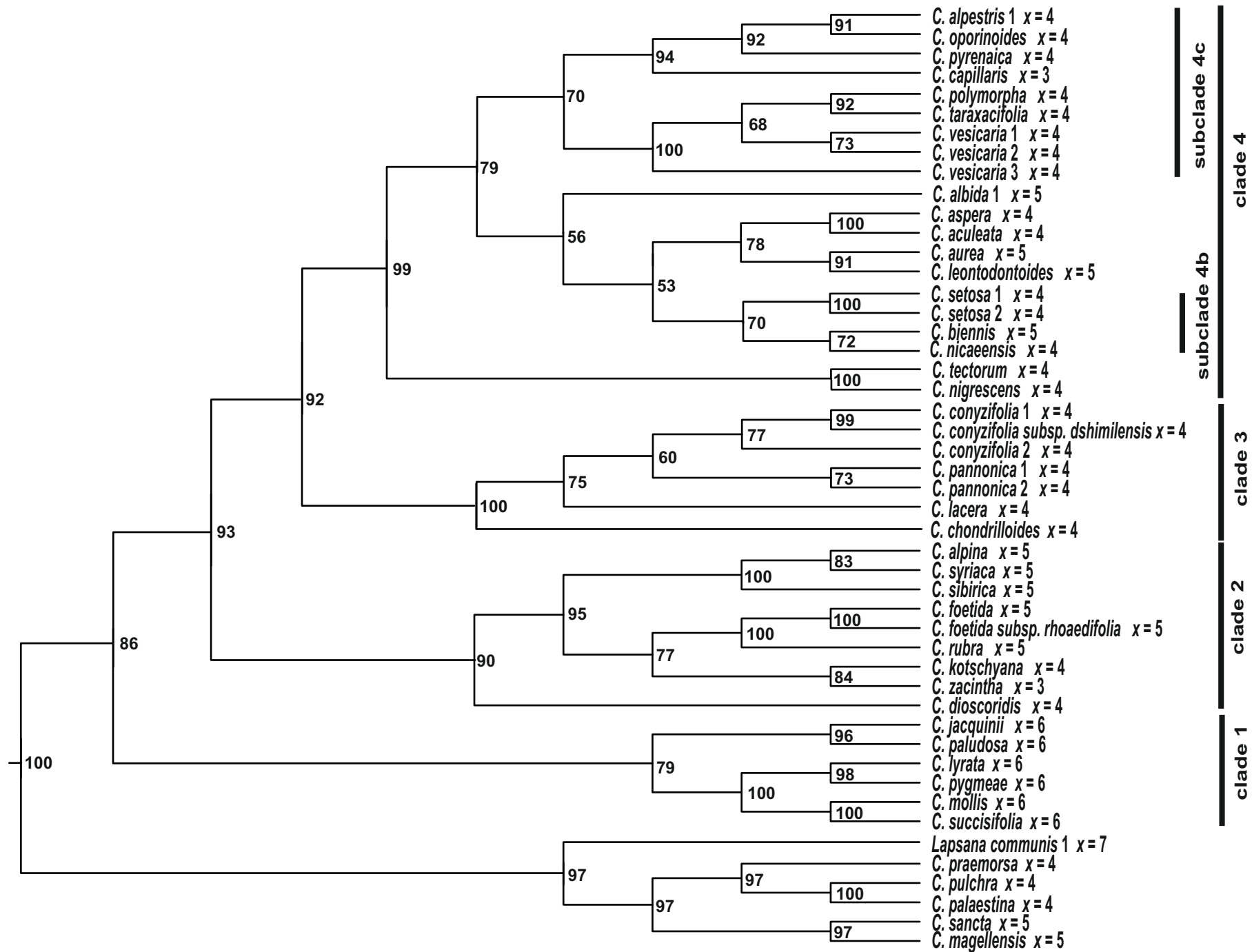

Lagoseris

*Crepis sensu stricto*

Supplement: Supplementary file 1 [file ijms-23-03643-s001.zip › Senderowicz et al. Figure S3.pdf]

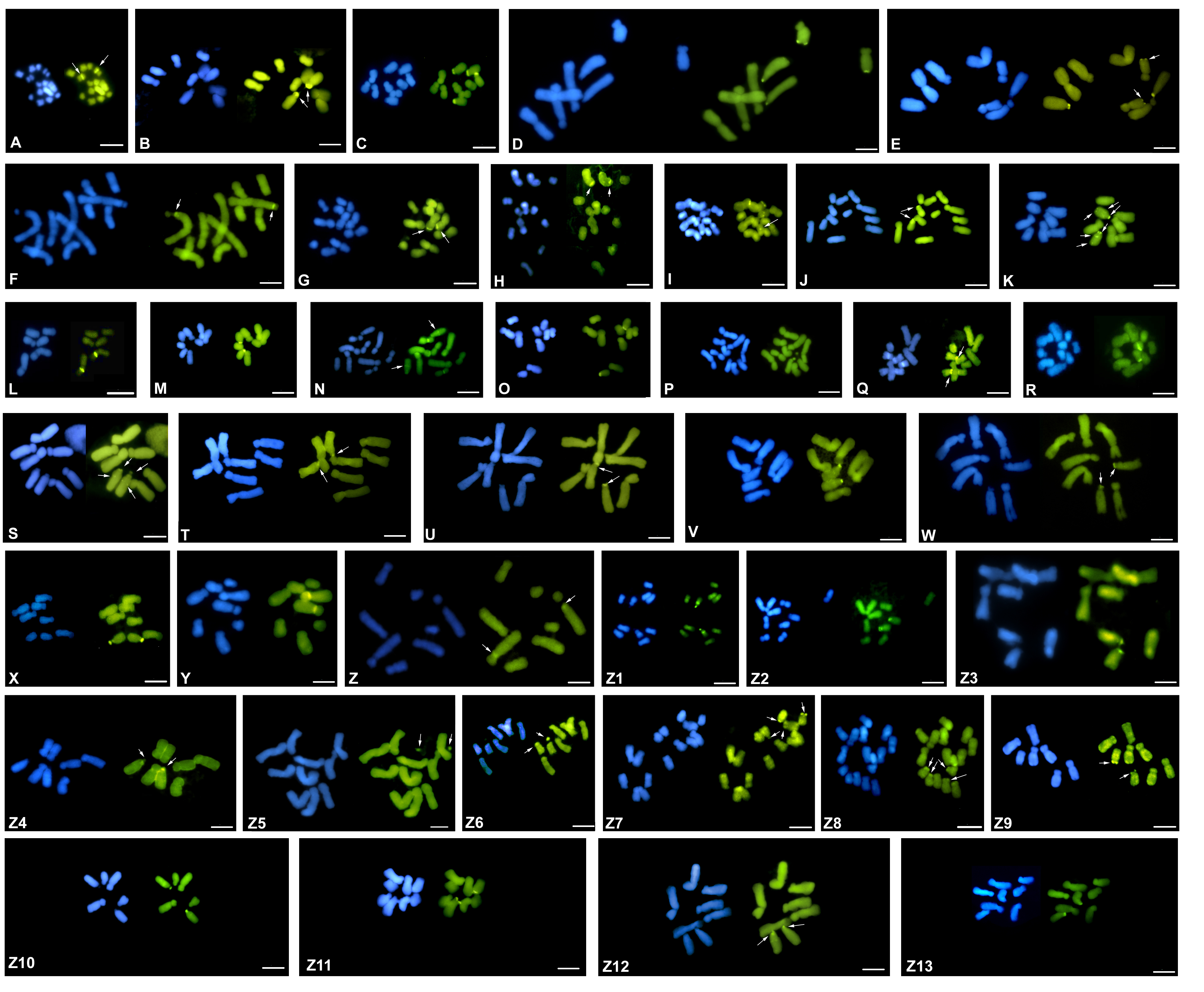

Supplement: Supplementary file 1 [file ijms-23-03643-s001.zip › Senderowicz et al. Figure S4.jpg]
